# Supplementary material for: Self acceleration from spectral geometry in dissipative quantum-walk dynamics
Source: Nat Commun. 2024 May 23;15:4381. doi: 10.1038/s41467-024-48815-y (PMC11116542; doi:10.1038/s41467-024-48815-y)
Supplement: Supplementary file 1 — Supplementary Information [file 41467_2024_48815_MOESM1_ESM.pdf]

SUPPLEMENTARY INFORMATION FOR "SELF ACCELERATION FROM SPECTRAL GEOMETRY IN DISSIPATIVE QUANTUM-WALK DYNAMICS"

In this Supplementary Information, we provide some technical and experimental details.

Supplementary Note 1 - Derivation of the Floquet operator U and the effective Hamiltonian

As mentioned in the main text, in the one-dimensional quantum walk, the Floquet operator reads $U = S_x M_x R(\theta)$. As all the operators can be expressed by Pauli matrices, the time-evolution operator can be also written as

$$\begin{aligned} U(k) &= d_0(k)\sigma_0 + i d_x \sigma_x + i d_y(k)\sigma_y + i d_z(k)\sigma_z \\ d_0(k) &= \cos(\theta)\cos(-i\gamma_x + k), \\ d_x(k) &= \sin(\theta)\cos(-i\gamma_x + k), \\ d_y(k) &= -\sin(\theta)\sin(-i\gamma_x + k), \\ d_z(k) &= \cos(\theta)\sin(-i\gamma_x + k). \end{aligned} \tag{1}$$

The effective Hamiltonian is defined through the relation $U = e^{-iH}$, which in the quasi-momentum space takes the form

$$H = \int_{-\pi}^{\pi} \mathrm{d}\boldsymbol{k}\, [E(\boldsymbol{k})\mathbf{n}(\boldsymbol{k}) \cdot \sigma] \otimes |\boldsymbol{k}\rangle\langle \boldsymbol{k}|, \tag{2}$$

with

$$\mathbf{n}(k) = \frac{1}{\sin E(k)} \begin{pmatrix} -\sin(\theta)\cos(k - i\gamma_x) \\ \sin(\theta)\sin(k - i\gamma_x) \\ -\cos(\theta)\sin(k - i\gamma_x) \end{pmatrix}. \tag{3}$$

Here the quasienergies are given by $E_{\pm}(k) = \pm \arccos[\cos\theta \cos(k - i\gamma_x)]$, which are the eigenvalues of H.

Similarly, for the two-dimensional case, we have

$$\begin{aligned} \mathbf{n}(\boldsymbol{k}) &= \mathbf{n}(k_x, k_y) \\ &= \frac{1}{\sin E(k_x, k_y)} \begin{pmatrix} -\cos(k_x + k_y - i\gamma_x - i\gamma_y)\cos(\theta_2)\sin(\theta_1) - \cos(k_x + k_y - i\gamma_x + i\gamma_y)\cos(\theta_1)\sin(\theta_2) \\ \sin(k_x + k_y - i\gamma_x - i\gamma_y)\cos(\theta_2)\sin(\theta_1) - \sin(k_x + k_y - i\gamma_x + i\gamma_y)\cos(\theta_1)\sin(\theta_2) \\ -\sin(k_x + k_y - i\gamma_x - i\gamma_y)\cos(\theta_2)\cos(\theta_1) - \sin(k_x + k_y - i\gamma_x + i\gamma_y)\sin(\theta_1)\sin(\theta_2) \end{pmatrix}, \end{aligned} \tag{4}$$

where the quasienergy is

$$E(k_x, k_y) = \pm \arccos\left[\theta_1 \cos(k_y - i\gamma_y - k_x + i\gamma_x) - \left(\frac{\pi}{2} - \theta_2\right)\cos(k_x - i\gamma_x + k_y - i\gamma_y)\right]. \tag{5}$$

Supplementary Note 2 - Correspondence between spectral topology and self-acceleration in one-dimensional lattices

1. *Single-band lattice models*

For the most exemplary class of NH lattice models, namely those featuring a single band with a point gap and no symmetry (see for example [1]), there is a one-to-one correspondence between non-trivial spectral point gap topology and self-acceleration, namely the following theorem 1 holds:

In any single-band NH lattice model, the early-time acceleration a_x of the center-of-mass wave packet under initial single-site excitation of the lattice is proportional to the spectral area $\mathcal{A}$ enclosed by the complex PBC energy spectrum $H(k)$ in the complex plane, namely

$$a_x = \frac{2}{\pi}\mathcal{A}. \tag{6}$$

The proof of such a general theorem was given in the earlier theoretical paper [2] and it is therefore not repeated here. Note that the above result clearly provides a universal correspondence between self-acceleration and spectral topology in generic one-band NH lattice systems without any special symmetry. Note also that, since the initial excitation condition is independent of the Hamiltonian $H(k)$, the dynamical probing method provides a universal tool for testing the spectral topology of the system that does not require any *a priori* knowledge of the system Hamiltonian.

2. Many-band lattice models

The photonic quantum walk setup used in our experiments is inherently a two-band system, and thus it is important to extend theorem 1 above to the multiband case. To this aim, let us consider an M-band NH lattice system, so that each unit cell of the lattice comprises M sublattice sites. Let us indicate by $a_n(t)$, $b_n(t)$, $c_n(t)$, ... the amplitudes of the wave function at the M sites in the n-th unit celll of the lattice. Let $H(k)$ be the $M \times M$ Bloch Hamiltonian of the system, and let us indicate by $E_\beta(k)$ and $(A_\beta(k), B_\beta(k), C_\beta(k), ...)^T$, the eigenvalues and corresponding eigenvectors of $H(k)$ $(\beta = 1, 2, ..., M)$, respectively. Specifically, we have

$$E_\beta(k) \begin{pmatrix} A_\beta(k) \\ B_\beta(k) \\ C_\beta(k) \\ ... \end{pmatrix} = H(k) \begin{pmatrix} A_\beta(k) \\ B_\beta(k) \\ C_\beta(k) \\ ... \end{pmatrix} \tag{7}$$

with $\beta = 1, 2, ..., M$. Clearly, there is some arbitrariness in the choice of $A_\beta(k)$, $B_\beta(k)$, ..., since they are defined up to an arbitrary complex factor that may depend on the Bloch wave number k. After a suitable choice of such a multiplication factor, it can be readily shown that the amplitudes $A_\beta k)$, $B_\beta(k)$, ... can be chosen to satisfy the following conditions

$$|A_\beta(k)|^2 + |B_\beta(k)|^2 + |C_\beta(k)|^2 + ... = 1 \ , \ \ A_l^*(k) \frac{dA_\beta}{dk} + B_\beta^*(k) \frac{dB_\beta}{dk} + ... = 0. \tag{8}$$

for any $\beta = 1, 2, 3, ..., M$. The first condition in Eq.(8) corresponds to wave function normalization, whereas the second condition in Eq.(8) corresponds to the gauge choice such that the diagonal elements of Berry connection vanish. Since the Hamiltonian $H(k)$ is non-Hermitian, the eigeneneriges $E_\beta(k)$ and the corresponding eigenvectors are not necessarily single-valued functions of the Bloch wave number k, i.e., if we continuously follow the change of the eigenvalue $E_\beta(k)$, as k continuously changes from $k = -\pi$ to $k = \pi$ along the Brillouin zone, we can have a flip of eigenvalues and eigenvectors at the end of the cycle. Only after M cycles are the initial eigenenergy and eigenstate retrieved. The nontrivial energy-surface topology is typically associated with the fact that the cycle encloses one or more exceptional points (see for instance [3–6]). We say that the multi-band system has a trivial energy-surface topology whenever there is no eigenenergies and eigenevector flipping after one cycle (when k continuously change from $k = -\pi$ to $k = \pi$ along the Brillouin zone).

The mean position of a wave packet at time t in the physical space is given by

$$\langle n(t) \rangle = \frac{\sum_n n(|a_n(t)|^2 + |b_n(t)|^2 + |c_n(t)|^2 + ...)}{\sum_n (|a_n(t)|^2 + |b_n(t)|^2 + |c_n(t)|^2 + ...)}. \tag{9}$$

The following theorem 2 can be stated:
For a given band index α of the lattice $(\alpha = 1, 2, ..., M)$, let us prepare the system at the initial time $t = 0$ in the state

$$a_n(0) = \frac{1}{2\pi} \int_{-\pi}^{\pi} A_\alpha(k) \exp(ikn) \ , \ b_n(0) = \frac{1}{2\pi} \int_{-\pi}^{\pi} B_\alpha(k) \exp(ikn) \ , \ c_n(0) = \frac{1}{2\pi} \int_{-\pi}^{\pi} C_\alpha(k) \exp(ikn) \ ... \tag{10}$$

where the Bloch eigenevector amplitudes $A_\alpha(k)$, $B_\alpha(k)$, ... are assumed to satisfy Eq. (8). Then, if the system has a trivial energy-surface topology, the early-time acceleration a_x of the wave packet center of mass is given by

$$a_x = \frac{2}{\pi} \mathcal{A}_\alpha, \tag{11}$$

where $\mathcal{A}_\alpha$ is the area enclosed by the curve $E_\alpha(k)$ in the complex plane.

Proof. To prove theorem 2, let us first observe that the most general solution to the time-dependent Schrödinger equation in the multi-band lattice system reads

$$\begin{pmatrix} a_n(t) \\ b_n(t) \\ c_n(t) \\ ... \end{pmatrix} = \sum_{\beta=1}^{M} \int_{-\pi}^{\pi} dk F_\beta(k) \begin{pmatrix} A_\beta(k) \\ B_\beta(k) \\ C_\beta(k) \\ ... \end{pmatrix} \exp[ikn - iE_\beta(k)], \tag{12}$$

where the spectral amplitudes $F_\beta(k)$ are determined by the initial excitation of the system at time $t = 0$. Let us assume that the initial state is the one defined by Eq.(10), which corresponds to the choice $F_\beta(k) = (1/2\pi)\delta_{\alpha,\beta}$ for the spectral amplitude. Hence one has

$$\begin{pmatrix} a_n(t) \\ b_n(t) \\ c_n(t) \\ ... \end{pmatrix} = \frac{1}{2\pi} \int_{-\pi}^{\pi} dk \begin{pmatrix} A_\alpha(k) \\ B_\alpha(k) \\ C_\alpha(k) \\ ... \end{pmatrix} \exp[ikn - iE_\alpha(k)]. \tag{13}$$

From Eq.(13), one can calculate the terms $\sum_n |a_n(t)|^2$, $\sum_n |b_n(t)|^2$, $\sum_n |c_n(t)|^2$,.. and $\sum_n n|a_n(t)|^2$, $\sum_n n|b_n(t)|^2$, $\sum_n n|c_n(t)|^2$,... . For example, one has

$$\sum_n |a_n(t)|^2 = \frac{1}{4\pi^2} \int_{-\pi}^{\pi} dk \int_{-\pi}^{\pi} dk' A_\alpha(k) A_\alpha^*(k') S(k-k') \exp\left[-itE_\alpha(k)t + iE_\alpha^*(k')t\right], \tag{14}$$

where we set

$$S(k-k') = \sum_n \exp[i(k-k')n]. \tag{15}$$

It follows that, in the range of variability of k and k',

$$S(k-k') = 2\pi\delta(k-k'), \tag{16}$$

and thus

$$\sum_n |a_n(t)|^2 = \frac{1}{2\pi} \int_{-\pi}^{\pi} dk |A_\alpha(k)|^2 \exp[2E_{I\alpha}(k)t], \tag{17}$$

where $E_{I\alpha}(k)$ is the imaginary part of the complex energy $E_\alpha(k)$. Similar expressions are found for $\sum_n |b_n(t)|^2$, $\sum_n |c_n(t)|^2$, ..., namely

$$\sum_n |b_n(t)|^2 = \frac{1}{2\pi} \int_{-\pi}^{\pi} dk |B_\alpha(k)|^2 \exp[2E_{I\alpha}(k)t]\ , \ \sum_n |c_n(t)|^2 = \frac{1}{2\pi} \int_{-\pi}^{\pi} dk |C_\alpha(k)|^2 \exp[2E_{I\alpha}(k)t]\ , \ ... \tag{18}$$

Taking Eq. (8) into account, from Eqs. (17) and (18), one then obtains

$$\sum_n (|a_n(t)|^2 + |b_n(t)|^2 + |c_n(t)|^2 + ...) = \frac{1}{2\pi} \int_{-\pi}^{\pi} dk \exp[2E_{I\alpha}(k)t]. \tag{19}$$

Let us now calculate $\sum_n n|a_n(t)|^2$. One has

$$\sum_n n|a_n(t)|^2 = -i\frac{1}{4\pi^2} \int_{-\pi}^{\pi} dk' \int_{-\pi}^{\pi} dk A_\alpha(k) A_\alpha^*(k') \exp\left[-itE_\alpha(k)t + iE_\alpha^*(k')t\right] . \frac{\partial S(k-k')}{\partial k} \tag{20}$$

After integration by parts and taking into account that $E_\alpha(\pi) = E_\alpha(-\pi)$ and $A_\alpha(\pi) = A_\alpha(-\pi)$ owing to the trivial energy surface topology, one readily obtains

$$\sum_n n|a_n(t)|^2 = \frac{i}{2\pi} \int_{-\pi}^{\pi} dk \left(A_\alpha^* \frac{dA_\alpha}{dk} - it|A_\alpha(k)|^2 \frac{dE_\alpha}{dk} \right) \exp[2E_{I\alpha}(k)t]. \tag{21}$$

Similar expressions are found for $\sum_n n|b_n(t)|^2$, $\sum_n n|c_n(t)|^2$, ..., namely

$$\sum_n n|b_n(t)|^2 = \frac{i}{2\pi}\int_{-\pi}^{\pi} dk \left(B_\alpha^* \frac{dB_\alpha}{dk} - it|B_\alpha(k)|^2 \frac{dE_\alpha}{dk} \right) \exp[2E_{I\alpha}(k)t],$$
$$\sum_n n|c_n(t)|^2 = \frac{i}{2\pi}\int_{-\pi}^{\pi} dk \left(C_\alpha^* \frac{dC_\alpha}{dk} - it|C_\alpha(k)|^2 \frac{dE_\alpha}{dk} \right) \exp[2E_{I\alpha}(k)t], \tag{22}$$
$$\dots \dots$$

Taking into account of Eq. (8), from Eqs.(21) and (22) one obtains

$$\sum_n n(|a_n(t)|^2 + |b_n(t)|^2 + |c_n(t)|^2 + ...) = \frac{t}{2\pi}\int_{-\pi}^{\pi} dk \frac{dE_\alpha}{dk} \exp[2E_{I\alpha}(k)t]. \tag{23}$$

Using Eqs. (19) and (23), the evolution of the wave packet center of mass [Eq.(9)] finally reads

$$\langle n(t) \rangle = \frac{t\int_{-\pi}^{\pi} dk \frac{dE_\alpha}{dk} \exp[2E_{I\alpha}(k)t]}{\int_{-\pi}^{\pi} dk \exp[2E_{I\alpha}(k)t]}, \tag{24}$$

which holds at any given instant t. In particular, in the early time dynamics with $t \to 0$, the above expression can be written as a series expansion in powers of t. At the lowest order, one obtains

$$\langle n(t) \rangle \simeq \frac{t^2}{\pi} \mathcal{A}_\alpha, \tag{25}$$

where we have set

$$\mathcal{A}_\alpha = \int_{-\pi}^{\pi} dk \frac{dE_{R\alpha}}{dk} E_{I\alpha}(k), \tag{26}$$

and $E_{R\alpha}(k)$, $E_{I\alpha}(k)$ are the real and imaginary parts of the energy $E_\alpha(k)$, respectively. Clearly, $\mathcal{A}_\alpha$ corresponds to the area enclosed by the energy $E_\alpha(k)$ in the complex plane as k traverses the Brillouin zone. Equation (25) shows that, at early time, the wave packet center of mass displays an accelerated motion with an acceleration $a_x = 2\mathcal{A}_\alpha/\pi$, which thus provides a dynamical measure of the spectral geometry of the α-th lattice band. This concludes the proof of theorem 2.

It should be mentioned that, as compared to the single-band case, in multi-band lattices the initial state preparation of the system [Eq.(10)] requires some knowledge of the Hamiltonian and can be in practice challenging. However, for narrow-band systems where the bands are spaced in complex energy plane by wide gaps, such as when the detuning of the on-site potentials at sites a, b, c, ... are much larger than the hopping amplitudes, in the appropriate basis the required initial excitation basically reduces to single-site excitation as in the single-band case, thus relaxing the condition of prior knowledge of the Hamiltonian. In our experiment, we are dealing with a two-band system and the narrow-band regime is attained for a coin angle θ close to $\pi/2$. The corresponding initial excitation of the system is very simple, as discussed in the Method section, and we can accurately estimate the spectral areas from wave-packet self-acceleration measurements.

Supplementary Note 3 - Experimental scheme

We implement quantum walks by employing a time-multiplexed configuration, where the external spatial modes are encoded through discretized temporal shifts, and the internal coin-state degrees of freedom are encoded using photonic polarizations (Fig. 1). With this experimental arrangement, we successfully carried out one-dimensional and two-dimensional quantum walks in the same experimental platform under various configurations, as demonstrated in the following.

The overall architecture is that of a fiber network, through which attenuated single-photon pulses with a wavelength of 808 nm and a pulse width of 88ps are sent, with each full cycle around the network representing a discrete time step. Laser pulses are attenuated by neutral density filters, which effectively reduce the energy of the laser pulses to the single-photon level at the detection stage. This step aims to maintain an average photon number per pulse below 2.6×10^{-4} to minimize the probability of multi-photon events. The input intensity of the initial laser pulse can be increased when we aim to obtain amplitude distribution of quantum walks after larger numbers of steps.

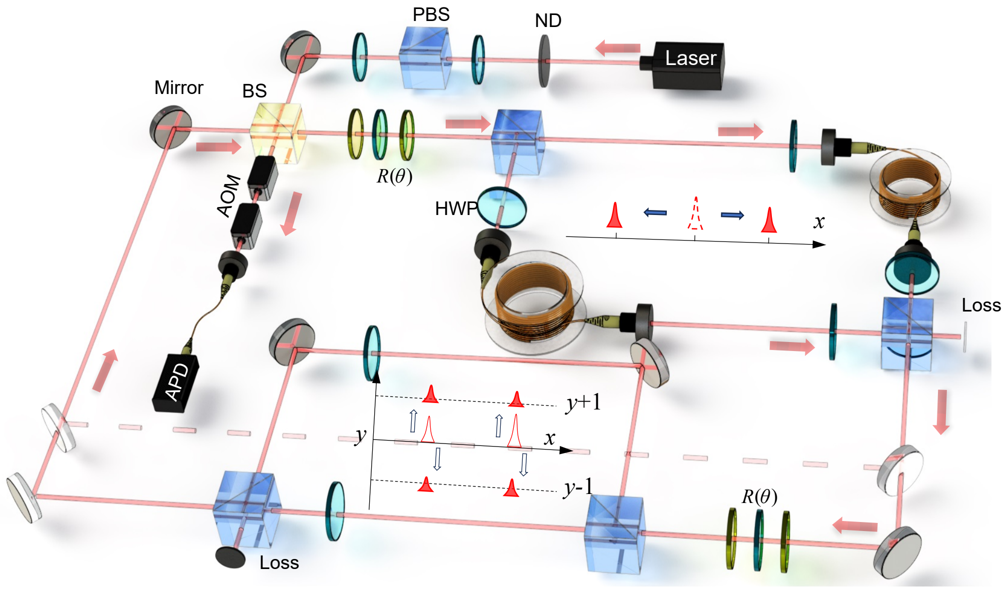

Supplementary Figure 1. **A time-multiplexed implementation of the two-dimensional photonic quantum walk.** The process involves splitting photonic wave packets using a polarizing beam splitter (PBS) and guiding them through a pair of single-mode fibers (SMF) to achieve a temporal step in the x direction. Similarly, a temporal step in the y direction is achieved using another two-PBS loop in free space. At each step, partial photons are coupled out and directed towards avalanche photodiodes (APDs) for polarization resolving detection of their arrival times. ND: neutral density filter; AOM: optical switch acousto-optic modulator.

The coin states $\{|0\rangle, |1\rangle\}$ are encoded in the photon polarizations $\{|H\rangle, |V\rangle\}$. The single-photon pulse act as the walker, exhibiting distribution across multiple temporal modes. This is achieved by building path-dependent time delays into the four different paths (labeled $x \pm 1$ and $y \pm 1$) in Fig. 1 within the network. Specifically, for two-dimensional quantum walks, the temporal modes were separated by two different time scales: 80ns in the x-dimension and 4.83ns in the y-dimension. For the one-dimensional quantum walk, the temporal modes were solely distributed along the x-dimension.

To implement the two-dimensional quantum walk, we rewrite the time-evolution operator as $U^t = e^{\gamma_x \gamma_y t} U_E^t$, where

$$U_E^t = M_y^{'} S_y R(\theta_2) M_x^{'} S_x R(\theta_1) \tag{27}$$

with $M_i^{'} = e^{-|\gamma_i|} M_i, i = x, y$.

The coin state is initialized after single-photon pulses pass through a polarizing beam splitter (PBS) and a half-wave plate (HWP). Subsequently, the photons are coupled in and out from a time-multiplexed configuration via a beam splitter (BS) with a reflectivity of 3%. The coin operator $R(\theta_{1(2)})$ is implemented by the sandwich-type set of wave plates (QWP-HWP-QWP), where QWP is the abbreviation of quarter-wave plate. To implement the shift operators, PBSs separate the photons with different polarizations and direct them into the two-fiber loop (S_x) or the free-space Mach-Zehnder interferometer (S_y). Specifically, the difference between two distinct fiber lengths (270m and 287.03m) are used to realize the polarization-dependent time delay 80ns in the x-dimension. The corresponding time difference in the y direction is 4.83ns, which is introduced by a 1.61m free space path difference of the free-space Mach-Zehnder interferometer. Importantly, the coherence of single-photon pulses is inherently preserved by the interference condition required for the single-particle quantum walk.

To implement the loss operation $M_x^{'}$, two HWPs are introduced into each fiber loop. The ones at the input and output ends of the fiber are also used to keep the polarizations of the single-photon pulses unchanged. For $\gamma_x > 0$, we adjust the angle of the HWP in the $x+1$ path satisfying $\cos\frac{\theta}{2} = e^{-2\gamma_x}$, the part of photons $1 - e^{-4\gamma_x}$ are transmitted by the second PBS, and leak out of the setup. For $\gamma_x < 0$, we set the angle of the HWP on the $x-1$ path to satisfy $\cos\frac{\theta}{2} = e^{2\gamma_x}$ and the part of photons $1 - e^{4\gamma_x}$ subsequently leak out of the setup. The loss operator $M_y^{'}$ is realized with the same method.

Arrival time is recorded by avalanche photodiodes (APDs), aided by an acoustic-optical modulator (AOM) that functions as an optical switch to eliminate unwanted pulses. The time-resolved pulses within the window are recorded

and translated to the corresponding spatial position of the walker. We measure the probability distribution of quantum walks

$$P(x,y,t)=\frac{|\langle\psi(x,y,t)|\psi(x,y,t)\rangle|}{\sum_{x,y}|\langle\psi(x,y,t)|\psi(x,y,t)\rangle|}=\frac{N(x,y,t)}{\sum_{x,y}N(x,y,t)}, \tag{28}$$

where $N(x,y,t)$ is the total photon number at the position (x,y) after a t-step evolution.

In our experimental setup, the loss of photons is primarily attributed to losses incurred by various optical elements. Even in the case of a unitary quantum walk, the overall efficiency of our round-trip single-loop is approximately 0.71. The overall efficiency is derived by multiplying the transmission rates of each optical component employed in the round trip, which include the transmission rate of the beam splitter (~ 0.97), the efficiency of collecting photons from free space to fiber (~ 0.80), and the transmission rates of all other optical components (~ 0.91). Thus, we have $0.71\simeq 0.97\times 0.80\times 0.91$. Besides, the measurement duration for a specific time step is approximately one hour, limited by the stability of our experimental setup.

Supplementary Note 4 - Initial state preparation and reconstruction of the center-of-mass motion

As discussed in the main manuscript, the initial excitation of the system should correspond to an equally-weighted superposition of Bloch eigenstates in a given lattice band. In our experiment, we cannot directly encode such an initial state since it contains excitation of both odd and even lattice sites, which is unfeasible with our setup. To overcome such a limitation, it is important to note that at each step, wave packets characterized by odd or even positions in the initial state do not interfere during the evolution process. This occurs because the evolved state exclusively occupy either even or odd positions when progressing to step t. We can take advantage of such a major property to split our experiment in two steps, where the initial excitation occupies either the even or odd lattice sites, and then reconstructing the wave packet dynamics exploiting the linearity of the system. To illustrate our strategy, let us consider the two-dimensional quantum walk as an example. We divided the experiment into two distinct parts, and we rewrite the initial state as

$$|\psi(0)\rangle=\left|\psi^1(0)\right\rangle+e^{\gamma_x-\gamma_y}\left|\psi^2(0)\right\rangle, \tag{29}$$

where

$$\left|\psi^1(0)\right\rangle=|1\rangle\otimes|0,0\rangle,\left|\psi^2(0)\right\rangle=|0\rangle\otimes|-1,1\rangle.$$

The evolved state is then $|\psi(t)\rangle=U^t|\psi(0)\rangle=U^t\left|\psi^1(0)\right\rangle+e^{\gamma_x t-\gamma_y t}U^t\left|\psi^2(0)\right\rangle$. Thus, the wave packet center of mass is given by

$$x_{CM}(t)=\frac{\sum_{x,y}\left[x\left|\langle\psi^1(x,y,t)|\psi^1(x,y,t)\rangle\right|+e^{2t(\gamma_x-\gamma_y)}x\left|\langle\psi^2(x,y,t)|\psi^2(x,y,t)\rangle\right|\right]}{\sum_{x,y}\left[\left|\langle\psi^1(x,y,t)|\psi^1(x,y,t)\rangle\right|+e^{2t(\gamma_x-\gamma_y)}\left|\langle\psi^2(x,y,t)|\psi^2(x,y,t)\rangle\right|\right]} \tag{30}$$

and

$$y_{CM}(t)=\frac{\sum_{x,y}\left[y\left|\langle\psi^1(x,y,t)|\psi^1(x,y,t)\rangle\right|+e^{2t(\gamma_x-\gamma_y)}y\left|\langle\psi^2(x,y,t)|\psi^2(x,y,t)\rangle\right|\right]}{\sum_{x,y}\left[\left|\langle\psi^1(x,y,t)|\psi^1(x,y,t)\rangle\right|+e^{2t(\gamma_x-\gamma_y)}\left|\langle\psi^2(x,y,t)|\psi^2(x,y,t)\rangle\right|\right]}. \tag{31}$$

One-dimensional quantum walks can also be realized with our setup by simply removing the free-space Mach-Zehnder interferometer. The probability distribution is obtained

$$P(x,t)=\frac{|\langle\psi(x,t)|\psi(x,t)\rangle|}{\sum_x|\langle\psi(x,t)|\psi(x,t)\rangle|}=\frac{N(x,t)}{\sum_x N(x,t)}, \tag{32}$$

where $N(x,t)$ is the total photon number at the position x after a t-step evolution.

Similarly, the chosen initial state can be written as

$$|\psi(0)\rangle=e^{\gamma_x}\left|\psi^1(0)\right\rangle+\left|\psi^2(0)\right\rangle, \tag{33}$$

where

$$\left|\psi^1(0)\right\rangle=|1\rangle\otimes|-1\rangle,\left|\psi^2(0)\right\rangle=|0\rangle\otimes|0\rangle. \tag{34}$$

The center of mass of the normalized involved state is

$$n_{CM}(t)=\frac{\sum_x \left(e^{2t\gamma_x} x\left|\langle\psi^1(x,t)|\psi^1(x,t)\rangle\right|+x\left|\langle\psi^2(x,t)|\psi^2(x,t)\rangle\right|\right)}{\sum_x \left(e^{2t\gamma_x}\left|\langle\psi^1(x,t)|\psi^1(x,t)\rangle\right|+\left|\langle\psi^2(x,t)|\psi^2(x,t)\rangle\right|\right)}. \tag{35}$$

Therefore, we perform two individual evolutions with different initial states, which finally enable us to reconstruct $x_{CM}(t)$, $y_{CM}(t)$, and $n_{CM}(t)$.

[1] Brunelli, M. *et al.* Restoration of the non-Hermitian bulk-boundary correspondence via topological amplification, *SciPost Phys.* **15**, 173 (2023).
[2] Longhi, S. Non-Hermitian skin effect and self-acceleration. *Phys. Rev. B* **105**, 245143 (2022).
[3] Heiss, W. D. The physics of exceptional points. *J. Phys. A* **45**, 444016 (2012).
[4] Longhi, S. Floquet exceptional points and chirality in non-Hermitian Hamiltonians, *J. Phys. A: Math. Theor.* **50**, 505201 (2017).
[5] Miri, M.-A. *et al.*, Exceptional points in optics and photonics. *Science* **363**, eaar7709 (2019).
[6] Longhi, S. *et al.*, Complex Berry phase and imperfect non-Hermitian phase transitions. *Phys. Rev. B* **107**, 085122 (2023).
